# Ultrasound of the Gallbladder—An Update on Measurements, Reference Values, Variants and Frequent Pathologies: A Scoping Review

**DOI:** 10.3390/life15060941

**Published:** 2025-06-11

**Authors:** Claudia Lucius, Barbara Braden, Christian Jenssen, Kathleen Möller, Michael Sienz, Constantinos Zervides, Manfred Walter Essig, Christoph Frank Dietrich

**Affiliations:** 1 Outpatient Department of Gastroenterology, IBD Centre Helios Klinikum Buch, 13125 Berlin, Germany; claudia.lucius@gmx.de; 2 Medical Department B, University Hospital Münster, 48149 Munster, Germany; barbara.braden@ukmuenster.de; 3 Department for Internal Medicine, Krankenhaus Märkisch Oderland, 15344 Strausberg, Germany; c.jenssen@khmol.de; 4 Brandenburg Institute for Clinical Ultrasound (BICUS) at Brandenburg Medical University, 16816 Neuruppin, Germany; 5 Medical Department I/Gastroenterology, SANA Hospital Lichtenberg, 10365 Berlin, Germany; k.moeller@live.de; 6 St. Benedict Ndanda Hospital, Internal Medicine, Ndanda P.O. Box 3, Tanzania; sienzjesaja@gmail.com; 7 CZMH Limassol Medical Physics and Dosimetry Services Ltd., Limassol, Cyprus; c.zervides@czmh.org; 8 Inselspital, University Hospital of Bern, 3010 Bern, Switzerland; 9 Department Allgemeine Innere Medizin (DAIM), Kliniken Hirslanden Beau Site, Salem und Permanence, 3013 Bern, Switzerland

**Keywords:** reference values, gallbladder, gallbladder wall, hydrops, gallbladder polyp, measurements, diameters, ultrasound, sonography, gallbladder disease, gallbladder emptying, examination standard

## Abstract

**Objective:** We aimed to provide an update on ultrasound measurements of the gallbladder with studies focusing on measurement techniques, reference values, and influencing factors. Anatomical anomalies and common pathological findings are discussed together with their clinical impact. **Methods**: A literature search was performed for ultrasound studies in healthy subjects. Relevant data published between 2010 and March 2025 were extracted and evaluated. Possible clinical implications are discussed. **Results**: Many factors influence gallbladder size and wall thickness, as the gallbladder is a highly functional organ. Diabetes and obesity have been proven to increase gallbladder volume and wall thickness. A normal gallbladder wall should be echogenic with one layer and a thickness < 3 mm. Gallbladder size is variable and can achieve values above 10 × 4 × 4 cm, especially with increasing age. Gallbladders with maximal diameters below 3.5 cm are referred to as micro-gallbladders. Calculating gallbladder volume is reserved for special issues, achieving the best inter- and intra-observer variability with the ellipsoid formula. Clinical relevance and work-up of common pathological findings like wall thickening, gallbladder polyps, and stones are discussed.

## 1. Introduction

The knowledge of reference values is crucial to distinguish physiological variations from pathological processes and, therefore, subsequently, for the clinical management of patients [1]. However, the measured values should not be seen in isolation but in the overall context of the clinical question, the patient’s history, laboratory values, and findings in the other organ systems. The image storing and documentation of measurements and normal findings should be part of the quality assurance in imaging [2,3].

This paper seeks to deliver a comprehensive review of the published literature, offering evidence-based insights into gallbladder (GB) sonographic measurements, examination techniques, and the spectrum of normal values. It delves into congenital variations, common pathological findings, and their potential clinical implications, supported by illustrative examples. Furthermore, the analysis extends to the influence of key demographic and physiological factors—such as age, gender, body constitution, and ethnicity—on gallbladder morphology and function, highlighting their relevance in diagnostic accuracy and clinical decision-making.

## 2. Materials and Methods

The literature on reference values in abdominal ultrasound (US) was reviewed based on three German-language publications from 2010 to 2012 by Sienz et al. [4,5,6], which will not be repeated but complemented with the published literature from 2010 until March 2025. In a series of papers, the use of measurements and the knowledge of reference values regarding other organ systems are currently updated [3,7,8,9,10,11,12].

### 2.1. Search Strategy

The PubMed Database was systematically searched for entries from 1 January 2010 until 23 March 2025 using: (“gall bladder” OR gallbladder OR cholecyst*) AND (ultrasound [title/abstract] OR ultrasonography [title/abstract] OR sonography [title/abstract] OR sonographic* [title/abstract]) AND (measurement OR volume OR diameter OR size OR lumen OR length OR “reference value” OR “normative value” OR “cut-off value”). In this way, 1049 entries were identified in PubMed (final search date: 23 March 2025).

### 2.2. Study Selection

Two of the authors independently reviewed titles and abstracts for eligibility. Animal studies, studies related only to pediatric cohorts (0–14 years), editorials, letters to the editors, duplicates, articles not referring to the gallbladder, articles including only measurements of pathologic conditions of the gallbladder, and articles only including non-US imaging modalities were excluded. Articles already included in Sienz et al.’s reference list were evaluated separately. They were included partially, as the review was published in German language and not all clinical implications were discussed [5]. Extensive cross-checking of the reference list of the retrieved articles was also performed. Disagreements regarding eligibility were resolved by discussion and consensus among all authors.

### 2.3. Data Extraction

**Figure 1:**
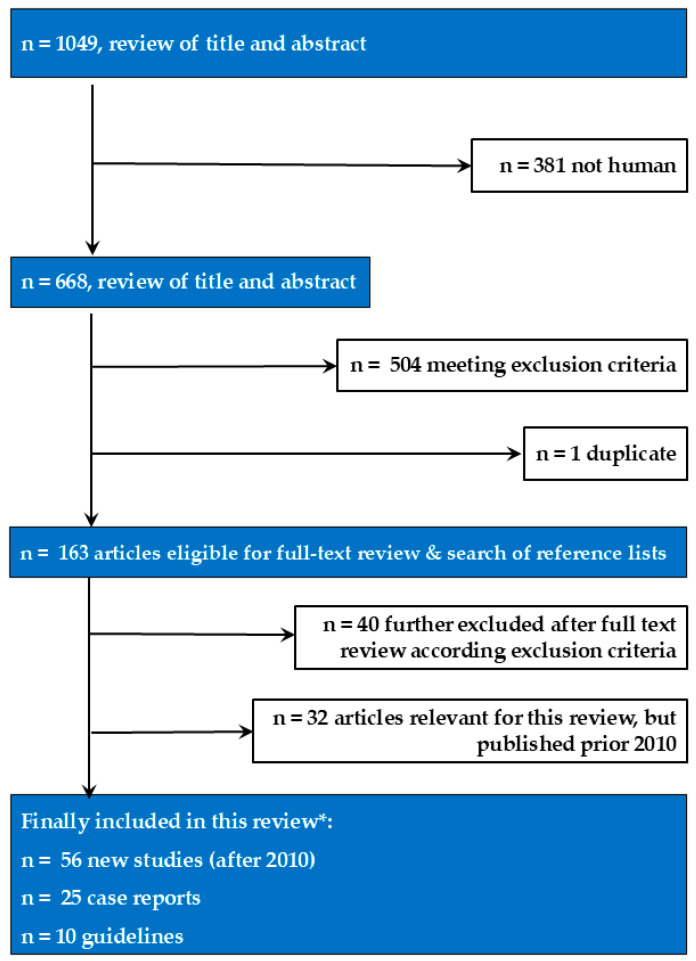
Flow chart describing search strategy and selection of studies included in this review. * Further references with important content were included from 2010 and earlier when not evaluated in the review by Sienz et al. [5] or recent reviews concerning additional clinical settings.

Data were extracted based on the year of publication and evaluated parameters (gallbladder wall, size, volume), topic (congenital changes, pathological findings), and imaging method used for assessment (e.g., transcutaneous US, endoscopic US). For search results, see the flow chart (Figure 1).

### 2.4. Indications for Sonographic Assessment of the Gallbladder

Ultrasound of the GB, encompassing the lumen, wall, and surrounding structures, is primarily indicated for evaluating right upper quadrant abdominal complaints, aiming to detect bile stones, cholecystitis, and potential complications. Furthermore, it is valuable in cases of pathological liver function tests and elevated enzymes indicative of cholestasis. Important differential diagnoses are diseases affecting the right costodiaphragmatic sinus, like pleural effusions, pneumothorax, or even lung diseases.

Beyond these typical indications, GB ultrasound provides significant information in patients with diabetes mellitus, metabolic syndrome, bile duct obstruction, and congestive (right) heart failure. It is also helpful in identifying congenital disorders, including cystic fibrosis, in the staging and monitoring of tumor patients, and post-surgery [13]. Typical biliary diseases include cholelithiasis and both common and rare inflammatory and neoplastic pathologies [14,15,16,17,18,19,20].

Regarding the management of pathological findings, endoscopic treatment options, such as endoscopy-guided interventions [21,22,23,24,25], and ultrasound-guided endoscopic and percutaneous treatment techniques [25,26,27,28,29,30,31], along with medical treatment and surgery, must be considered. The selection of the appropriate therapeutic approach depends on the nature and severity of the findings.

### 2.5. Examination Technique

**Figure 2:**
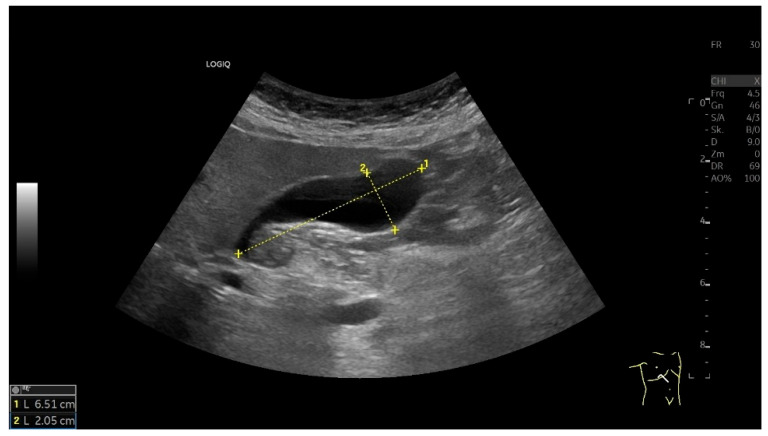
The GB is shown in its maximum longitudinal and transverse diameters and is measured in these two planes. The size is normal (<10 × 4 cm). The wall is echogenic and displayed with one layer (<3 mm). (Source: own collection, C.L.).

For correct measurement, the GB must be imaged in its maximum longitudinal extent and perpendicular to the longitudinal axis in its largest diameters in deep inspiration from a subcostal or, if not possible, from an intercostal transducer position [32,33,34] (Figure 2, Table 1).

The size, wall thickness, wall structure, and luminal contents of the gallbladder (GB) should be assessed, with routine measurements focusing on the maximal longitudinal and transverse diameters [13]. GB volume measurements are recommended only for functional studies [35]. While longitudinal diameter measurement may be challenging in angulated GBs, transverse diameter is usually measurable. Wall thickness is determined by measuring the maximal inner-to-outer distance perpendicular to the liver border from a right intercostal probe position [32,33,34]. Standard evaluations should include the anatomic site, wall thickening (extent and symmetry), mural layering, luminal contents (e.g., calculi, sludge), and intramural changes (e.g., cysts, echogenic foci) [13]. Examinations should be performed in the fasting state for optimal visualization. For mobile patients, standing or positional maneuvers like turning to the left side can aid in stone mobilization. Scanning in two planes through the entire GB, including the infundibulum, is essential (summarized in Table 1). Videos illustrating these techniques are available [36].

### 2.6. Prerequisites for Optimum Measurement

#### 2.6.1. Patient Preparation (Scheduled Examination)

A total of 6 to 8 h fasted. Beverages without sugar, gas, or milk are allowed up to 2 h before.

#### 2.6.2. Patient Position

- Supine position.
- A 15–30° left lateral oblique position.
- Seated or standing position.

#### 2.6.3. Transducer Type and Initial Position

Standard abdomen 2–10 MHz multifrequency curvilinear probe, held in the sagittal plane with the orientation marker pointing to cephalad. The probe is placed below the costal margin in the epigastric area and is swept along the right costal margin laterally. The GB comes into view in the right subcostal area at the medio-clavicular line. If the gallbladder cannot be found immediately, the interlobar fissure is sought ventral to the ramus principalis dexter of the portal vein in the subcostal horizontal transducer position. The gallbladder should be located in its extension. The transducer can then be turned clockwise into the longitudinal position. During these movements, the gallbladder can usually be visualized longitudinally in one of these positions. In addition, the gallbladder can be visualized from the intercostal transducer position.

## 3. Reference Values and Recommendations

### 3.1. Gallbladder Size (Length and Width)

The published literature on GB size is heterogeneous, making defining standard values difficult [4,5,6]. However, a micro-gallbladder is defined as a maximum diameter of <3.5 cm in the fasting patient and can frequently be observed in patients with cystic fibrosis and patients with chronic cholecystitis [37,38].

The longitudinal GB diameter is normally <10 cm [5]. The transverse GB diameters (width and depth) are approximately the same (<4 × 4 cm). Hydropic GBs > 10 × 4 × 4 cm are observed in some elderly patients without pathological underlying causes [5].

### 3.2. Gallbladder Volume

Dodds et al. showed a high accuracy of the ellipsoid method for volume determination [39]. The inter- and intra-observer variability for the ellipsoid method is reported to be below 10% [39,40,41]. However, the volumes measured with the ellipsoid method were significantly larger than those assessed with the cylinder summation method [42].

Recent studies stated volume measurements in a normal range between 27.2 ± 12.8 cm^3^ (range 6.96–108.1 cm^3^) in a Benin cohort [34], 24.2 ± 23.5 mL in a retrospective American cohort [32] or 27.2 ± 1.3 cm^3^ in a Nigerian study [33].

Treatment methods (oral litholysis and extracorporeal shock wave lithotripsy) that require volume measurements in a fasting condition and after a stimulus meal are rarely used today and not recommended, as long-term results and recurrence rates are disappointing.

In everyday clinical practice, especially in asymptomatic individuals, measuring two diameters (length and width) is sufficient.

### 3.3. Gallbladder Wall

The GB wall thickness depends on the GB’s contraction state. Due to its small size, the measurements vary in the literature from 1 to 2 mm [43] up to <3 mm (2.6 ± 1.6 mm) [32].

A GB wall diameter of >3 mm in fasting patients seems to be a reliable threshold value for clinical pathologies. According to Sienz et al., a normal value of ≤3 mm for the gallbladder wall thickness was identified [5]. These findings align with the cut-off used in CT diagnostics [44].

Reference values are summarized in Table 2.

### 3.4. Factors Influencing Interpretation

**Figure 3:**
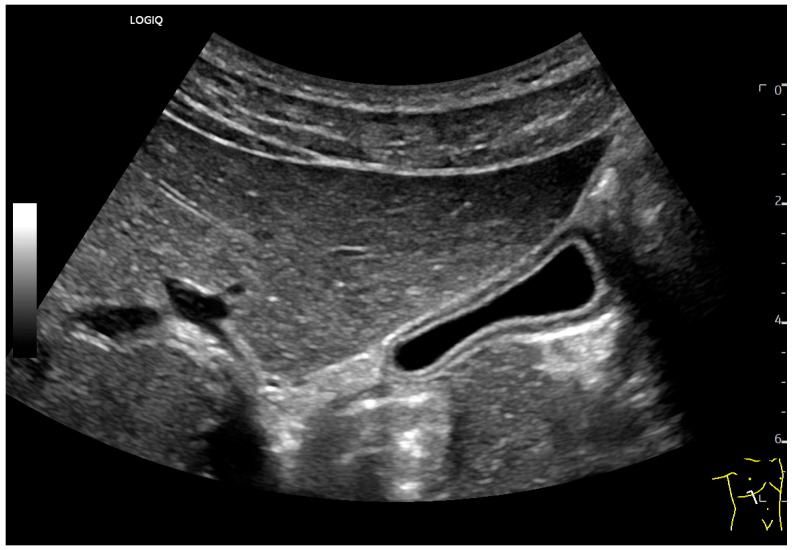
Small GB due to postprandial contraction. A three-layer wall is displayed. (Source: own collection, C.L.).

Due to the high functionality of the GB, the following factors often result in the same changes to all GB diameters, i.e., volume and wall. The GB diameters depend on the fasting timeline, with postprandial contraction as a functional sign (Figure 3).

Enlarged fasting GB volumes and reduced GB ejection fraction are described in patients with type 2 diabetes (DM2) [45,46]. A recent Nigerian case–control study confirmed significantly greater GB length, volume, and wall thickness in DM2 compared to controls [33].

Excessive body visceral fat and insulin resistance are found with higher BMI, which may predispose to GB motility disorders. A strong to moderate correlation between gallbladder wall thickening and BMI was noted [33,34,45,46].

In a Nigerian cohort evaluating women at 32 and 40 weeks of pregnancy, a higher BMI predisposed to larger GB volume, thereby possibly increasing the risk of bile stasis and gallstone formation during pregnancy [46]. Similar changes were found in a Danish study in pregnant women without further affection due to additional gestational diabetes [47]. In patients with intrahepatic cholestasis of pregnancy (ICP), significantly larger gallbladder basal volume and larger ejection fraction were detected compared to the control group. The authors suggest using this finding as additional assistance in diagnosing and assessing the severity of ICP [48].

A recent Nigerian study reported weak correlations between age, fasting blood sugar, and female gender [33]. At the same time, other studies found no significant differences related to gender, age, or body height [34].

Ethnic influence was suggested due to larger GB volumes in Nigerian studies compared to European and Asian populations, potentially linked to genetic, environmental, or nutritional factors [33]. However, other international studies reported no ethnic differences in GB measurements [34].

In patients with cholecystolithiasis, fasting and residual GB volumes are enlarged, resulting in higher postprandial GB volumes [40,49,50,51,52,53,54]. GB wall thickening is significant in those with gallstones or sludge [32,55].

Larger GB volumes are also found with distal bile duct obstruction (Courvoisier’s sign), autonomic neuropathy [46,56], gastrointestinal paresis [57], and several drugs (calcium channel blockers, opioids, anticholinergic acting drugs, hormones including estrogen and progesterone, non-steroidal antiphlogistic devices (NSAID), glucagon-like peptide 2-agonists [58]).

Same-day colonoscopy can influence imaging results. Shin et al. evaluated 89 Korean patients and found significantly smaller short-axis diameters of the gallbladder (<1.5 cm in 46%) correlating to decreased volumes in CT [59]. Smaller GB volumes are observed up to 2 h after radiologic examinations with contrast agents, e.g., for CT or urography, but not after MRI [60].

An overview of these different influencing factors is given in Table 3. In Table 4 recommendations for documentation and indications for measurement are summarized.

**Figure 4:**
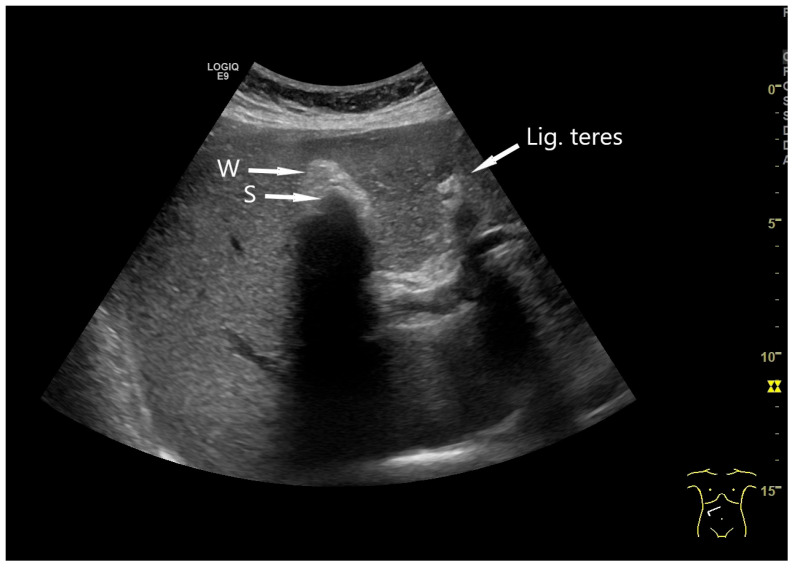
Shrunken gall bladder. Hyperechogenic thickened gallbladder wall (arrow, W). No free lumen is visible, only a single shadowing stone (arrow, S) with dorsal sound cancelation. The echogenic round ligament (arrow, Lig. teres) is shown, which divides the left liver lobe into its medial and lateral sections (Source: own collection, K.M.).

If the GB is not visible, conditions such as post-cholecystectomy, congenital anomalies, or a shrunken GB should be considered. A shrunken GB is the final state of chronic inflammatory and fibrotic alterations and is often asymptomatic. In shrunken GBs, echogenic stone reflexes surrounded by a hyperechoic irregular wall may be present (Figure 4).

## 4. Clinical Relevance of Common Pathological Findings

When assessing the GB, understanding pretest probabilities—particularly whether the patient is symptomatic (e.g., pain, fever, inflammation)—is crucial. Incidental findings like polyps, cholesterosis, and adenomyomatosis, seen in up to 12% of healthy individuals, require careful clinical evaluation [13].

### 4.1. Diffuse Gallbladder Wall Thickening

**Figure 5:**
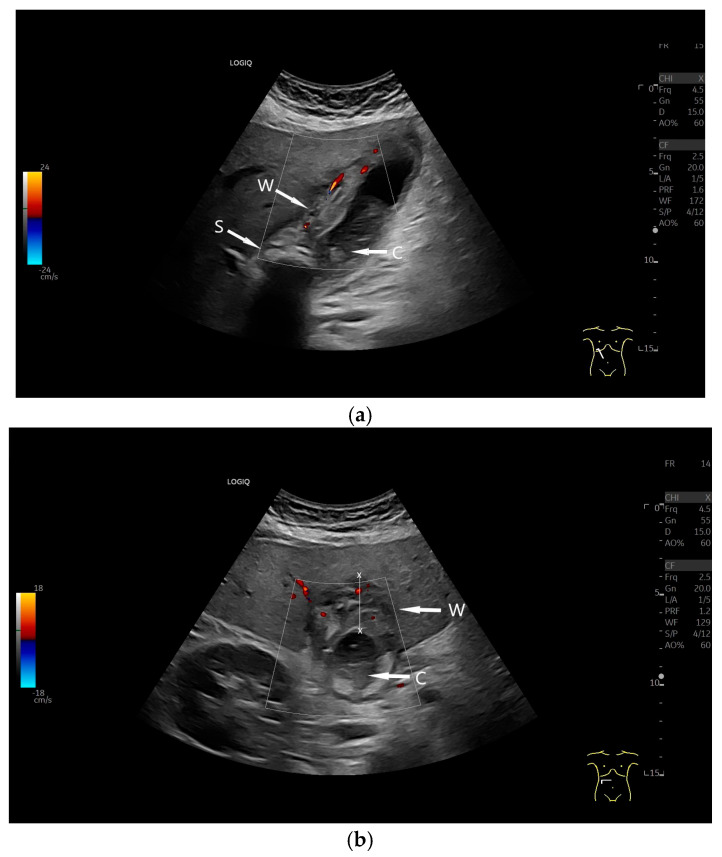
Acute cholecystitis. The GB wall (W) is significantly thickened and layered like onion skin in the context of right upper abdominal pain. Visual diagnosis of acute cholecystitis without measuring the GB wall. In the infundibulum there is a stone (S) with dorsal acoustic cancelation and echogenic content (C) in the lumen. Longitudinal (**a**) and transverse (**b**) section. (Source: own collection, K.M.).

A thickened, stratified GB wall, GB width > 30 mm, and localized pain from transducer pressure (Murphy’s sign) are indicative of acute cholecystitis in patients with acute right upper abdominal pain and elevated inflammatory markers [61] (Figure 5).

**Figure 6:**
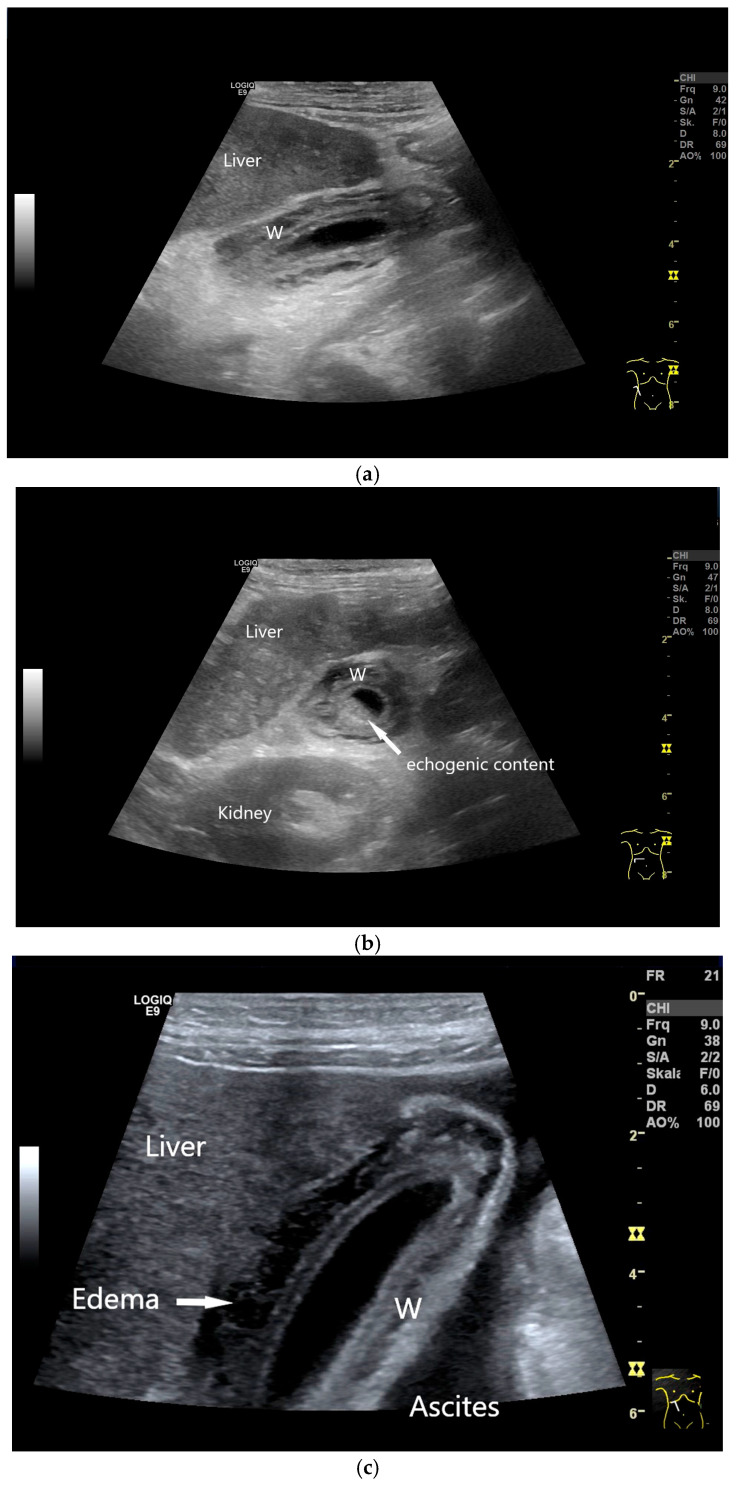
Decompensated liver cirrhosis with ascites. Flabby GB with thickened and stratified wall (W). Longitudinal (**a**) and transverse (**b**) sections. High-resolution transducer demonstrates wall edema (**c**). (Source: own collection, K.M.).

**Figure 7:**
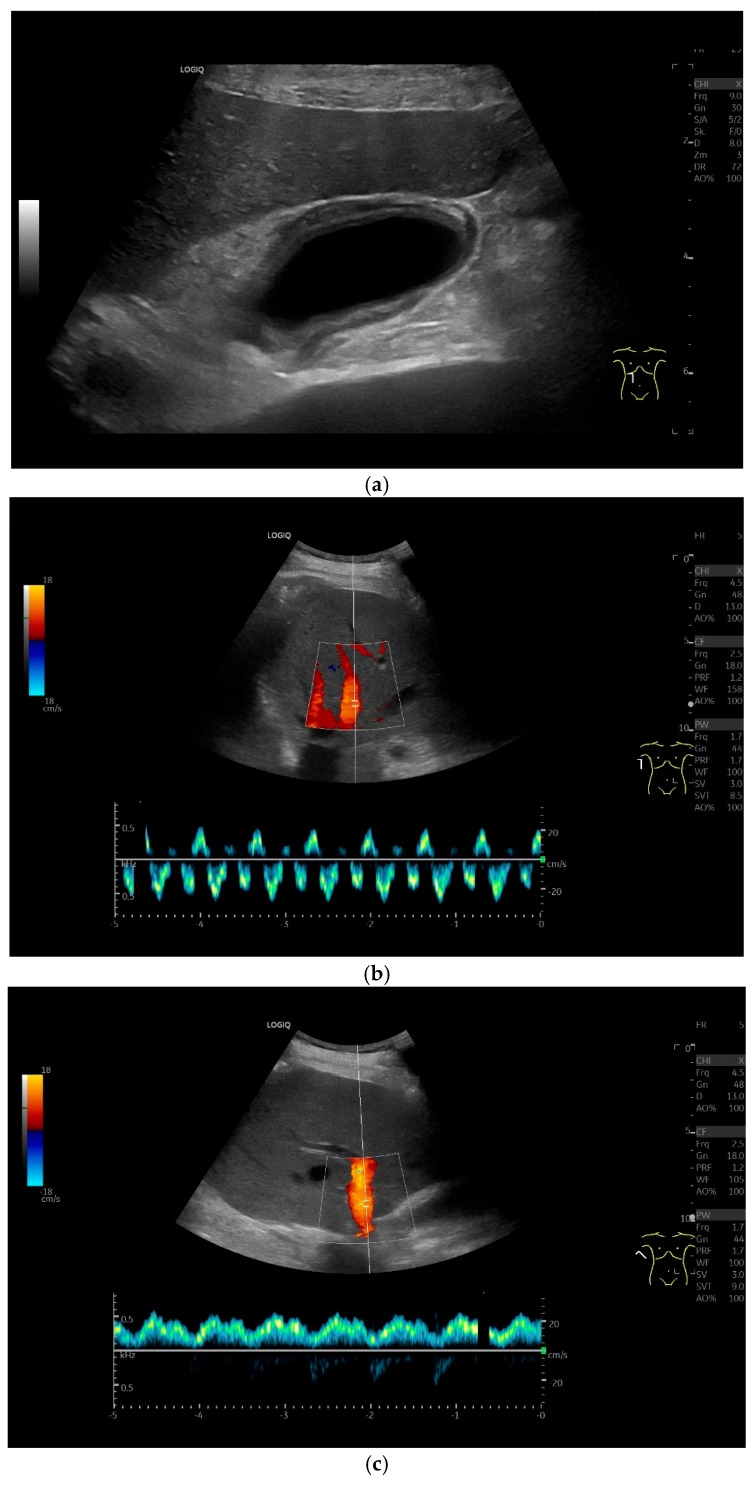
Congestive heart failure. Thickened and stratified GB wall (**a**), no stones, no clinical and laboratory signs of inflammation. Moderate pressure pain in the right upper abdomen due to liver congestion due to right heart failure. Pendulating flow in the hepatic veins (**b**) and strongly undulating flow in the portal vein (**c**) on Color Doppler imaging, indicating severe right heart insufficiency with trans-sinusoidal congestion. (Source: own collection, K.M.).

**Figure 8:**
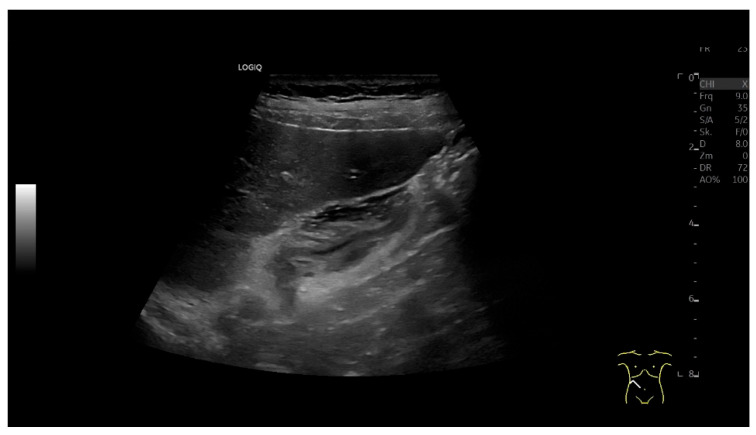
Acute viral hepatitis. Right-sided upper abdominal discomfort with isolated elevation of transaminases. The GB wall is thickened, and the lumen is empty. In addition, there was hepatosplenomegaly with lymph node enlargement in the hepatoduodenal ligament. Clinical context and lack of GB stones and negative Murphy’s sign are arguments against acute cholecystitis. (Source: own collection, K.M.).

Patients with hypoalbuminemia, ascites, liver cirrhosis, right heart failure, or acute hepatitis may present with a thickened, stratified gallbladder (GB) wall and a floppy GB shape [13] (Figure 6, Figure 7 and Figure 8). Wall edema with a compressed lumen does not always indicate acute cholecystitis; liver enlargement in acute hepatitis may cause capsule tension pain instead.

Accurate characterization of gallbladder (GB) wall thickening is critical for guiding treatment [62,63]. In acute hepatitis, wall thickening with a compressed/filled lumen is common (Figure 8), while acute cholecystitis typically shows stratified “onion-skin” layering and a fluid-filled lumen in cholecystolithiasis (Figure 5). The latter requires evaluation for perforation or hepatic penetration.

For a work-up of suspected acute cholecystitis, one imaging method is sufficient, according to Schuster et al. [64]. This recent American, multicenter, prospective study evaluated US, MRI, and CT in 861 patients, showing excellent agreement for diagnosing acute cholecystitis and, in particular, gallbladder wall thickness with only rare, maximal discordance of up to 1.02 mm. Using US as point-of-care-method in the emergency department may help to shorten the length of stay and time to treatment decision.

Concerning incidental findings of diffuse gallbladder (GB) wall thickening (>3 mm without a mass), Bird et al. [65] reported no clear follow-up recommendations based on Canadian and ACR guidelines from 2020. When gallbladder carcinoma cannot be excluded, further imaging with CT or MRI is recommended [66,67]. Newer studies confirmed a high accuracy for multiparametric MRI in differentiating benign from malignant gallbladder wall thickening in prior uncertain US or contrast-enhanced CT [68]. However, studies comparing MRI and contrast-enhanced US are scarce.

Contrast-enhanced ultrasound (US) and endoscopic US are additional tools for differentiating benign from malignant GB wall thickening [13,69,70,71].

### 4.2. Focal Gallbladder Wall Thickening

**Figure 9:**
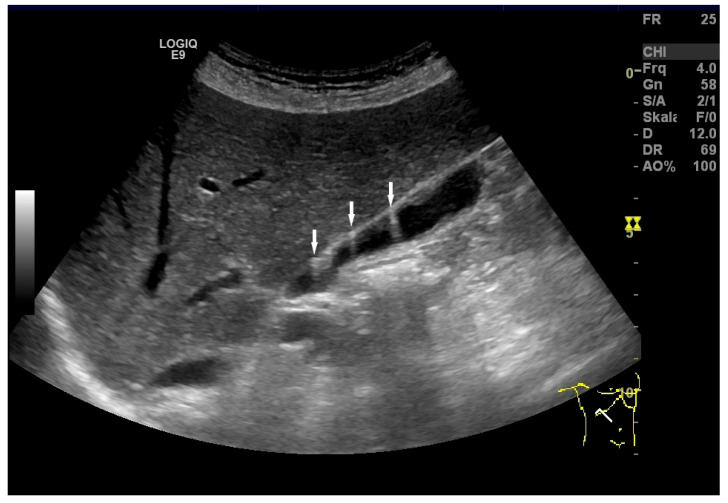
Cholesterolosis. Comet tail artifacts due to cholesterol crystals (arrows) within GB wall. (Source: own collection, C.L.).

**Figure 10:**
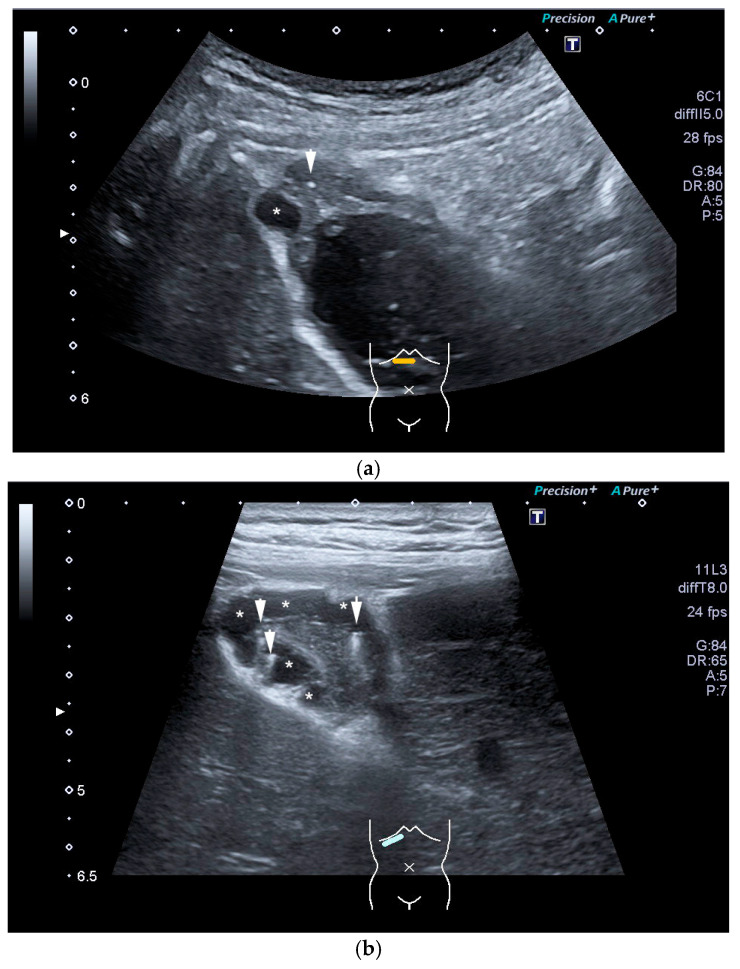
Adenomyomatosis. (**a**) Note echogenic wall thickening of fundus with a small intramural cyst (*) and tiny cholesterol crystals (arrow). (**b**) High-resolution transducer shows multiple intramural cysts (*) and comet tail artifacts (arrows). (Source: own collection, C.J.).

Determining whether the GB wall thickening is focal or diffuse is crucial. Adenomyomatosis, found in 1–9% of mostly asymptomatic older adults, involves mucosal hyperplasia, muscularis propria thickening, and cystic pockets (Rokitansky-Aschoff sinuses). On ultrasound, it appears as a thickened wall with tiny anechoic spaces and intracystic echogenic foci, causing characteristic comet tail artifacts [72,73] (Figure 9 and Figure 10).

A recent meta-analysis identified B-mode ultrasound (US) features distinguishing benign from malignant gallbladder (GB) wall thickening. Benign findings included echogenic foci, intact wall, and hypoechoic nodules, with sensitivities of 89%, 77%, and 66% and specificities of 86%, 51%, and 80%. Malignancy was associated with focal thickening and indistinct liver interface (sensitivities: 75% and 55%; specificities: 64% and 69%) [74]. The Gallbladder Reporting and Data System (GB-RADS) aids in risk stratification [75]. High-resolution US or endoscopic ultrasound (EUS) is recommended to enhance GB wall analysis [13,76].

### 4.3. Gallbladder Polyps

Gallbladder polyps vary in appearance, ranging from echogenic to hypoechoic, with homogeneous or inhomogeneous features. Unlike stones, they remain fixed at their base during positional changes, though polyps with longer stalks may shift slightly.

Differentiation between gallbladder polyps and biliary sludge may be challenging. Fine deposits of biliary sludge may occasionally simulate gallbladder wall polyps on ultrasound. A useful technique for distinguishing between the two is dynamic scanning, achieved by altering the patient’s position during the examination. Biliary sludge is typically mobile and detaches from the wall, whereas true polyps remain fixed [77].

**Figure 11:**
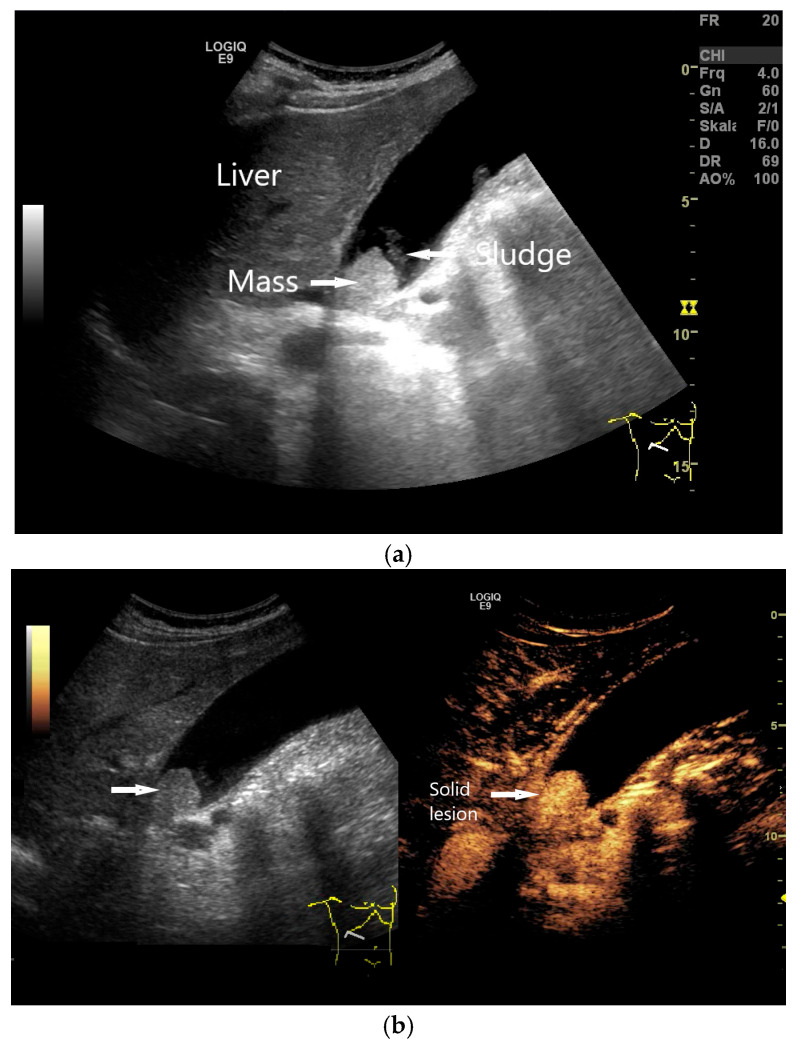
GB polyp. In a symptom-free patient, a 20 mm homogeneous mass (arrow) is found in the infundibulum of the GB (**a**), which is not hydropic. Some sludge swirls up. The lesion is constant in position on repositioning. In CEUS with 1.2 mL SonoVue, the mass is enhanced, indicating a solid lesion/polyp and excluding mass-forming sludge (**b**). Cholecystectomy is indicated. (Source: own collection, K.M.).

**Figure 12:**
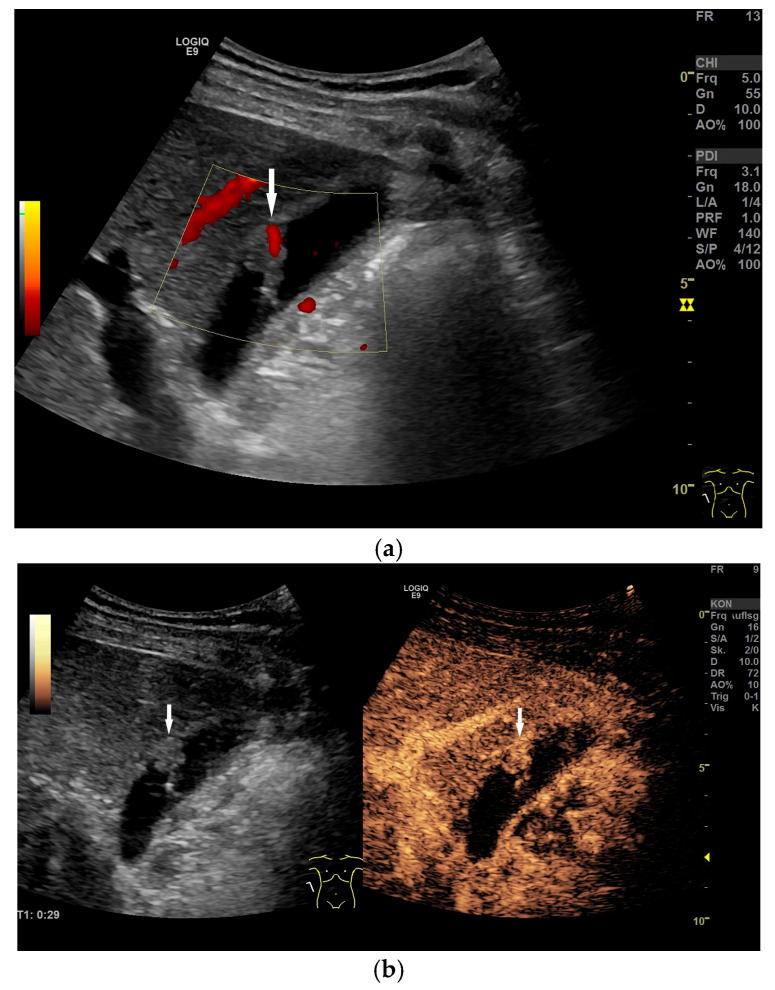
GB polyp: Irregularly shaped polyp of maximal size of 17 mm with a central vessel (arrow) on power Doppler imaging (**a**), proving that the finding is not an artifact. On CEUS, the polyp is enhanced (arrow) (**b**), excluding sludge. Cholecystectomy is indicated. (Source: own collection, K.M.).

The sensitivity for gallbladder polyp detection in transabdominal US is 84%, with a specificity of 96% [78]. Risk factors for malignancy are solitary polyp, size ≥ 10 mm, sessile polyp ≥ 4 mm, irregular surface, focal wall disruption, and wall thickening ≥4 mm [79]. US shows higher sensitivity compared to CT (93.5% vs. 66.1%) for predicting neoplastic lesions using the 10mm cut-off size [80]. In this retrospective Korean study, polyp sizes measured by CT and US in the same patients showed slightly higher values for US (11.4 ± 4.5 mm vs. 7.4 ± 4.9 mm in CT), most probably due to the general higher spatial resolution in US [80]. On high-resolution US, features of neoplastic polyps were a single lobular surface, central vessel, hypoechoic appearance, and hypoechoic foci [81] (Figure 11 and Figure 12). Especially in oncologic patients’ gallbladders, metastasis must be considered [82].

A polyp size of more than 1 cm was independently associated with a neoplastic appearance [81,83]. In patients with primary sclerosing cholangitis, even small polyps appear to harbor an increased risk of carcinoma. For this reason, cholecystectomy is recommended in this patient group regardless of size [84,85]. Patients’ age may be another factor to be considered. A Chinese multicenter study found the above-mentioned polyp criteria more important for middle-aged subjects. Localization in the fundus in younger and elderly people was an independent risk factor for neoplastic polyps [86].

No recommendation regarding the growth threshold is given [65,87]. When evaluating suspected growth especially of small polyps, it is important to be aware of possible measurement uncertainties. According to a recent Korean study, size changes in <1.9 mm seemed to be within the measurement error [88]. If the polyp grows to 10 mm or more during surveillance, a cholecystectomy is recommended [85].

No follow-up is recommended if the polyp is 5 mm or smaller and there are no risk factors according to the European guidelines [13,85] or <6 mm according to the American and Canadian guidelines [65,79]. An excellent algorithm for management and follow-up considering all these factors is given by Foley et al. within the European guidelines [85].

Besides obesity, metabolic steatotic liver disease has been shown as an independent risk factor for GB polyp development [89]. Therefore, increasing incidences can be assumed. The associated costs for surgery and follow-up imaging for management of the usually incidentally detected GB polyps should be considered when applying all these recommendations [90].

### 4.4. Gallstones

**Figure 13:**
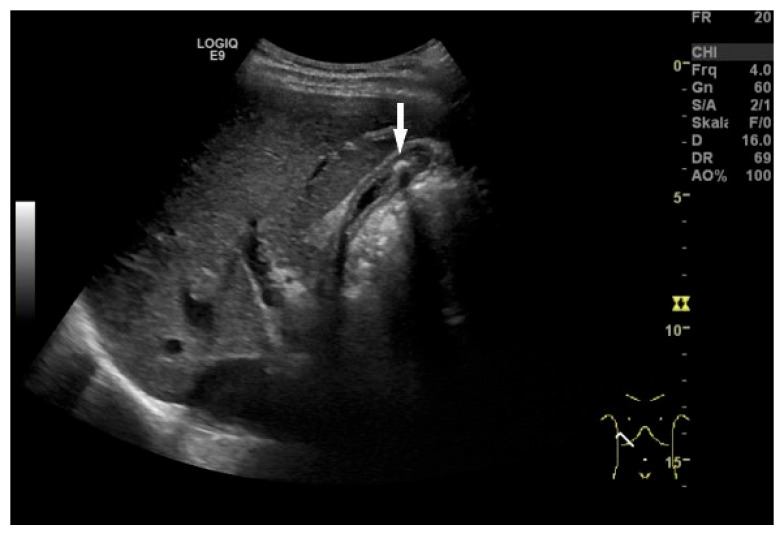
Cholecystolithiasis. Small postprandial contracted GB with postprandial stratification. There is a gallstone (arrow) in the lumen identified by the hyperechogenic dome and dorsal shadowing. (Source: own collection, K.M.).

**Figure 14:**
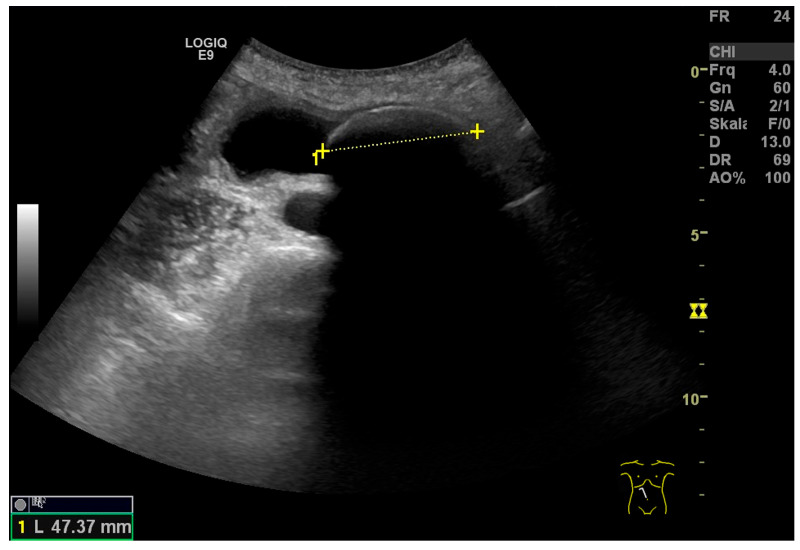
Large GB stone. Longitudinal section of GB with a 47 mm large stone (between markers). GB wall tender and inconspicuous. (Source: own collection, K.M.).

**Figure 15:**
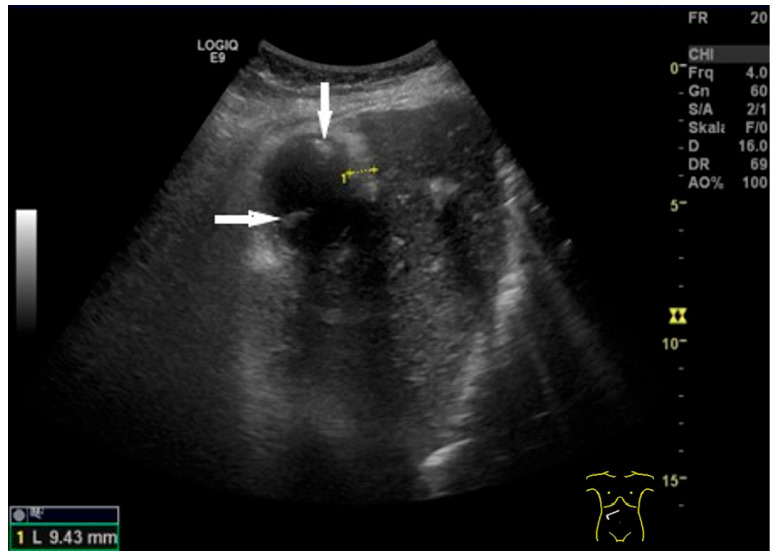
Chronic cholecystitis in cholecystolithiasis. Hyperechoic wall thickening (between markers). No postprandial stratification. Stones (arrows) in the lumen. (Source: own collection, K.M.).

Gallstones are a common incidental finding in adults. US is the method of choice for diagnosing gallstones with a sensitivity > 95% and a specificity of about 100% [91]. Gallstones present as a hyperechoic dome reflex with a dorsal acoustic shadow (Figure 13). Up to 20% of adults have gallbladder stones, and more than 20% of these people develop symptoms, mostly colics [92]. Gallstones > 3 cm in size are considered a risk factor for developing gallbladder carcinoma [93]. Cholecystectomy should be performed in these patients, even if they are asymptomatic (Figure 14). If gallstones lead to hyperechoic GB wall thickening this is referred to as chronic cholecystitis (Figure 15). This is probably due to intermittent obstruction of the cystic duct, leading to chronic inflammatory infiltration of the wall with subsequent fibrosis and reduced size. The final state is called a shrunken gallbladder (compare Figure 4). Pericholecystic inflammation is usually absent [13].

### 4.5. Gallbladder Hydrops

**Figure 16:**
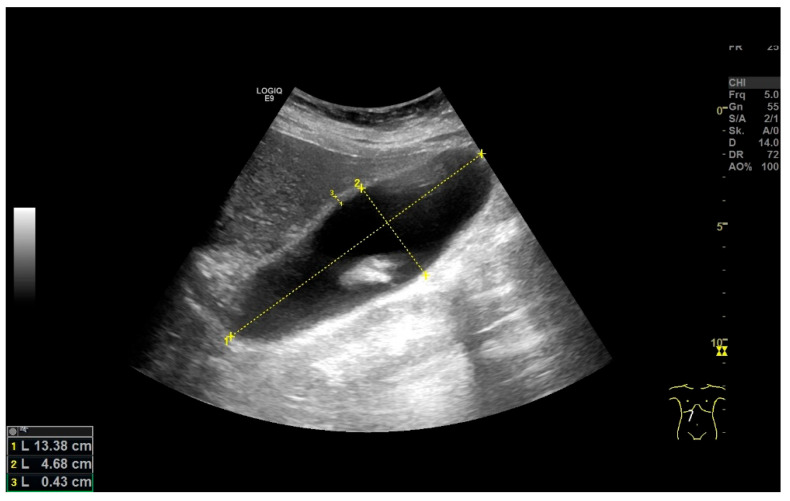
After the implantation of a fully coated metal stent in the common bile duct, a patient developed symptoms with an increase in inflammatory parameters. GB hydrops with a size > 10 × 4 cm (distance markers 1 and 2) and accompanying cholecystitis with a wall thickness > 4 mm (distance marker 3). (Source: own collection, K.M.).

GB hydrops can accompany acute cholecystitis, cystic duct stones, or neoplastic occlusions. GB hydrops due to obstruction in the bile duct system occur when the obstruction is distal to the insertion of the cystic duct (Figure 16).

## 5. Congenital Changes and Their Clinical Relevance

**Figure 17:**
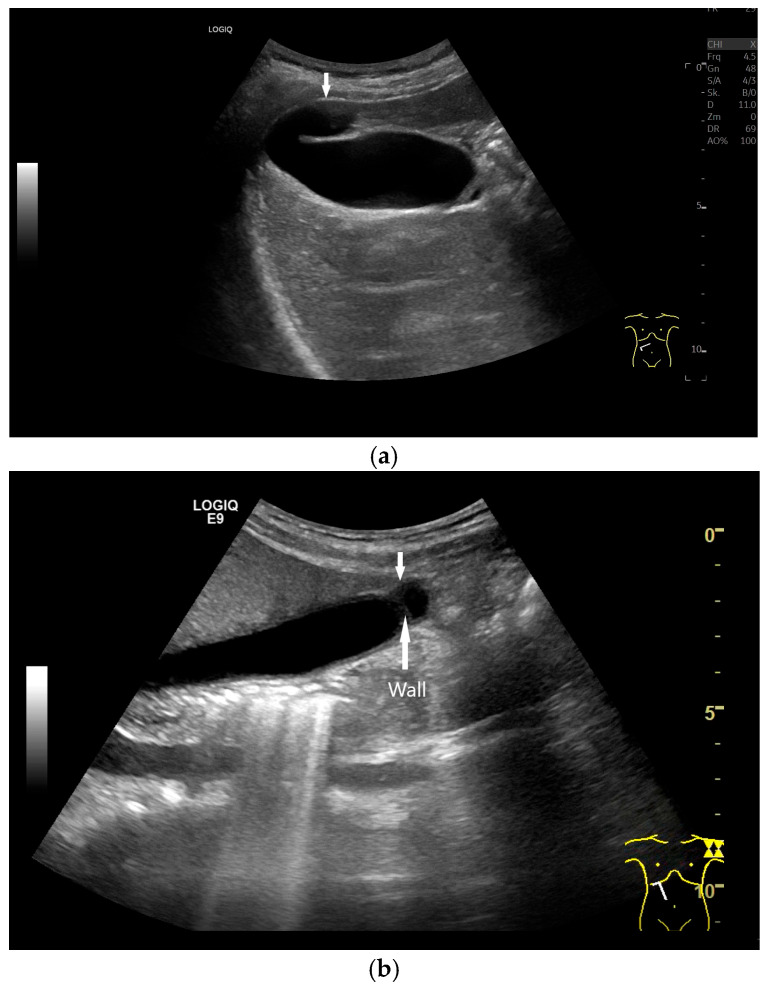
Phrygian cap. At the GB fundus, the Phrygian cap (arrow) is represented as a pointed bend (**a**). In another example, a second ostium appears on the fundus (arrow) (**b**). The wall (W) is visible as a boundary (arrow). It is not a cystic lesion but a section of a Phrygian cap. (Source: own collection, K.M.).

Due to the variability in the gallbladder (GB) shapes and sizes, intra-individual evaluation (e.g., size doubling or additional folds) is recommended but requires prior non-pathological ultrasound for comparison. Congenital GB anomalies, such as the Phrygian cap (Figure 17) and Hartmann’s pouch (Table 5), are common. While often asymptomatic, they may increase gallstone risk. Hartmann’s pouch is linked to cholecystolithiasis and can complicate cholecystectomy.

Congenital GB anomalies in number (agenesis, duplication, triple lumen GB) and size are very rare and may cause differential diagnostic problems (Table 6).

Finally, anomalies of anatomical location (Table 7) are often misinterpreted. Repeating questions about the clinical history (cholecystectomy? symptoms?) and variation in the patient’s and/or probe position may be helpful. In particular, variations in localization can be a challenge for surgical approaches.

**Figure 19:**
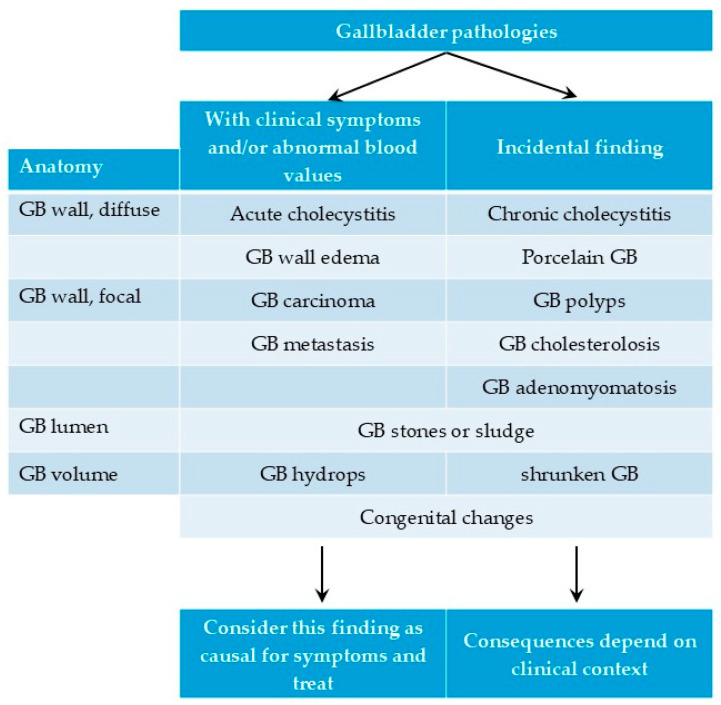
Overview of gallbladder pathologies depending on clinical context.

An overview of GB pathologies with their possible clinical impact is given in Figure 19.

## 6. Future Perspectives, Open Questions

Data from large registries will help to clarify the role of GB measurements in many diseases and conditions (e.g., obesity, COVID-19, IgG4, pregnancy, age). Deep learning and neural network methods are applied in many medical fields and sonographic imaging analysis, with some promising results [139,140,141]. Measurements of volume and wall thickening might be automated [142]. However, the translation into clinical practice has still to overcome many hurdles and needs prospective clinical evaluations.

## 7. Conclusions

A standardized GB examination should be performed under fasting conditions, utilizing the subcostal view for optimal visualization. Key measurements—including the maximal longitudinal diameter, transverse diameter, and wall thickness—should be meticulously recorded, alongside evaluating the wall structure. The GB lumen must be thoroughly assessed, with any luminal structures carefully described. In cases of acute symptoms, the examination can be conducted at any time; however, postprandial GB contraction should be considered when encountering a markedly small GB. GB volume measurements are reserved for functional studies to ensure diagnostic precision. Clinicians must remain vigilant regarding the wide range of congenital variations that may influence findings. All pathological observations should be meticulously documented and measured to facilitate accurate comparisons during follow-up evaluations. Importantly, the clinical significance of these findings must always be interpreted within the context of the patient’s individual circumstances to guide effective management and treatment.

## Figures and Tables

**Figure 18:**
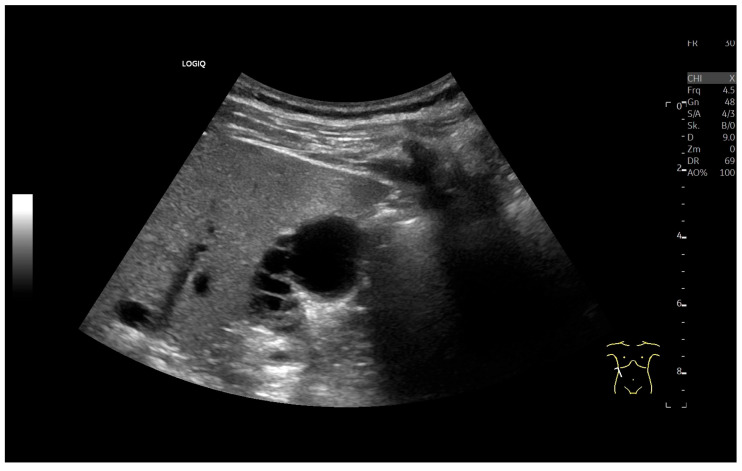
Multiseptated gallbladder in an asymptomatic patient. (Source: own collection, C.L., K.M.).

**Table 1:** What should you exercise when learning US?

Anatomical Structure	What Should I Do?
Gallbladder	Imaging of the GB in its maximum longitudinal extent in the longitudinal section at the level of the medio-clavicular line. Be aware of the infundibulum and scan it thoroughly. Use various transducer positions and body positions (supine, left-sided, also standing). Measurement of the following: Maximum longitudinal diameter; The transverse diameter; The thickness of the GB wall adjacent to the liver border.

**Table 2:** Overview of reference values of gallbladder measures. For details, see text.

Measured GB Structures	Reference Values
Length × width × depth	<10 × 4 × 4 cm [5,32,33,34].
Wall thickness	<3 mm [5,32,33,34]

**Table 3:** Overview of influencing factors. For study details, see text. Symbols describe changes of GB volume, wall or ejection fraction due to the corresponding factor ↑ = increased, ↓ = reduced, --= no influence.

Influencing Factor	GB Volume	GB Wall	GB Ejection Fraction
Postprandial state	**↓**	**↑**	
Type 2 diabetes	**↑**	**↑**	**↓**
High BMI		**↑**	
High BMI and pregnancy	**↑**		
Several drugs (e.g., NSAID, GLP2-agonists)	**↑**		
Same-day colonoscopy, urography, or other contrast agents	**↓**		
Gender, ethnicity, and age	--	--	--

**Table 4:** Recommended documentation and measurements.

Indication	Anatomical Structure
Routine examination	Gallbladder in two axes, GB wall without measurement
Defined clinical indications	GB wall measurement GB volume measurement (functional assessment)

**Table 5:** Congenital anomalies of the gallbladder shape.

Gallbladder Shape—Congenital Anomalies		
Nature of Changes	Description	Meaning
Phrygian cap [94,95,96,97,98,99] (Figure 17)	- GB is angled in the area of the fundus—either by folding or a septum. - Most common abnormal form with 1–7% prevalence. - Pseudo-duplication of the GB can occur in the presence of a Phrygian cap with an incidence of 0.025%.	- Can be missed if the GB is not assessed in several planes. - Can potentially lead to misdiagnosis of thickened GB wall or mistaken as liver lesion. - No significance unless gallstones are hiding there.
Hartmann’s gallbladder pouch [100,101,102,103]	- Hartmann’s pouch is an outpouching of the GB at the transition of the GB to the cystic duct. Prevalence varies from 4.7 to 52%. - Common finding in normal and pathologic GBs.	- Significantly associated with cholecystolithiasis. - Hartmann’s pouch stones encountered during laparoscopic cholecystectomy may hinder the safe dissection of the cystic pedicle.
Sigmoid gallbladder/Constriction with two pouches [13,104]	Described as two pouches with a narrow isthmus in between, like two GB in a line.	Differential diagnosis of a cystic lesion/tumor. Clinical relevance for surgery.
Multiseptated gallbladder [105,106,107,108] (Figure 18)	- Multiple septa of various sizes. “Honeycomb-like” appearance. - Rare and benign anomaly with <150 cases reported.	Differential diagnosis of multicystic tumor, lymphangiosis, xanthogranulomatous cholecystitis
Diverticula [13,109,110,111,112,113,114]	- Congenital or acquired. - Prevalence 0.001–0.2%.	- Differentiate true diverticula (all layers involved) and pseudodiverticula (secondary after partial perforation. - Risk of inflammation due to bile stasis and sludge formation.

**Table 6:** Congenital anomalies of number and size.

Gallbladder Anomalies of Number and Size		
Nature of Changes	Description	Meaning
Agenesia [13,115,116,117,118,119,120]	Non-displayable GB. Prevalence of 0.01–0.3% with a male-to-female ratio of 1:3. The incidence during autopsy was reported to be 0.035–0.3%.	Misdiagnosis of a shrunken GB and unnecessary surgery due to adjacent intestinal air that may be mistaken for concrements.
Hypoplasia/Micro-gallbladder [121,122]	Incomplete development of the embryonal GB bud. Very small GB.	Associate conditions such as cystic fibrosis, biliary atresia, cholangitis, neonatal hepatitis are reported. Differential diagnoses are postprandial contraction, chronic cholecystitis, choledochal cyst. Symptomatic patients benefit from laparoscopic cholecystectomy.
Duplication (Partial or complete) [96,99,101,121,123,124,125]	A duplicated GB may present bilobed, Y-shaped or V-shaped. Bilobed GBs have two completely divided cavities. Prevalence of 0.02–2%. Only 50% of cases with GB duplication are detected pre-operatively on conventional imaging.	Differential diagnoses are angled GB, choledochal cyst, Phrygian cap, GB diverticulum, adenomyomatosis. Diagnosis is easier when gallstones are present. Cholecystitis can affect one or both lumina.
Vesica fellea triplex [126]	Triple gallbladder resulting from incomplete regression of rudimentary bile ducts. It is a very rare condition: Between 1958 and 2022, only 21 cases were identified and published.	Increased risk of gallbladder metaplasia, dysplasia, and adenocarcinoma. There is an association between gastric and duodenal metaplasia with the potential for adenocarcinoma development.

**Table 7:** Congenital anomalies of location.

Gallbladder Location—Congenital Anomalies		
Nature of Changes	Description	Meaning
Left-sided gallbladder [121,127,128,129]	The GB is located on the left side of the ligamentum teres. There are three anatomic variants: Situs inversus; Left-sided ectopic gallbladder; Right-sided ligamentum teres with failure in the right lobe development. In US, it is represented as a cystic lesion ventral to the pancreas.	Often not detected until surgery. Differentiated surgical techniques. Higher incidence of common bile duct injury at cholecystectomy due to anomalies of the bile duct, portal vein, and other structures.
Intrahepatic gallbladder [121,130]	Completely surrounded by liver parenchyma, often with biliary stasis and cholelithiasis.	Acute cholecystitis may represent as hepatic abscess secondary to GB perforation. Preoperative diagnosis is important to avoid biliary injuries.
Suprahepatic gallbladder position [131,132,133]	- Positioned on lateral liver margin or subdiaphragmal. - Overlay by lung artifacts possible.	Association with other congenital changes in the right lobe of the liver is possible.
Floating gallbladder [134,135,136,137]	The gallbladder is suspended from the mesentery and can move freely. The gallbladder changes position during repositioning.	- Torsion with acute pain symptoms is possible. - Risk for acute cholecystitis.
Inside the lesser omentum [138]	Enclosed in the right free margin of the lesser omentum.	Possible complications in laparoscopic cholecystectomy.
